# Correlation between Sleep Bruxism, Stress, and Depression—A Polysomnographic Study

**DOI:** 10.3390/jcm8091344

**Published:** 2019-08-29

**Authors:** Joanna Smardz, Helena Martynowicz, Anna Wojakowska, Monika Michalek-Zrabkowska, Grzegorz Mazur, Mieszko Wieckiewicz

**Affiliations:** 1 Department of Experimental Dentistry, Wroclaw Medical University, 50-425 Wroclaw, Poland; 2 Department of Internal Medicine, Occupational Diseases, Hypertension and Clinical Oncology, Wroclaw Medical University, 50-556 Wroclaw, Poland

**Keywords:** bruxism, sleep bruxism, masticatory muscle activity, stress, depression, polysomnography

## Abstract

*Background and objectives:* Sleep bruxism is a common phenomenon that can affect approximately 13% of adult population. It is estimated that bruxism can be caused by three types of factors: biological, psychological, and exogenous. There are many scientific reports about the coexistence of bruxism, stress, and psychoemotional disorders. The aim of this study is to evaluate the possible correlation between occurrence of sleep bruxism and perceived stress and depressive symptoms. *Material and methods:* The material of this study consisted of 77 patients of Clinic of Prosthetic Dentistry operating at the Department of Prosthetic Dentistry, Wroclaw Medical University, Poland in which after using guidelines of the American Academy of Sleep Medicine probable sleep bruxism was fund. Patients then underwent video-polysomnography. Exposure to perceived stress was evaluated with Perceived Stress Scale-10 (PSS-10). Occurrence of depressive symptoms was evaluated with Beck’s Depression Inventory (BDI). *Results:* The analysis showed lack of statistically significant correlation between Bruxism Episodes Index (BEI) and Perceived Stress Scale–10 and Beck’s Depression Inventory scores (*p* = 0.64, *p* = 0.65; respectively), also when comparing study group (bruxers) and control group (non-bruxers) (*p* = 0.88, *p* = 0.77; respectively). *Conclusion:* Intensity of sleep bruxism was not statistically significantly correlated with self-reported perceived stress and depression. This issue requires further research.

## 1. Introduction

Bruxism is a phenomenon commonly associated with clenching and grinding the teeth. It is estimated that it occurs in 8–31% of the population without significant differences in relation to gender. It can be divided into awake bruxism (AB) and sleep bruxism (SB) [1,2,3]. Lobbezoo et al. in 2018 international consensus proposed two separated definitions of bruxism [1]. Awake bruxism is the activity of the masticatory muscles occurring during the waking period, which is characterized by sustained or repetitive contact between the teeth or/and stiffening or thrusting of the mandible and is not a movement disorder in otherwise healthy individuals [1]. Sleep bruxism is defined as the activity of masticatory muscles during sleep, which may be rhythmic (phasic) or non-rhythmic (tonic) and is not a movement disorder or a sleep disorder in otherwise healthy individuals [1]. The phrase “masticatory muscle activity” in both definitions indicates the potential clinical consequences of both types of bruxism. It is estimated that bruxism can be provoked by three groups of factors. Biological factors including neurotransmitters, genetic factors, and cortical arousals are the first group. Psychological factors such as stress sensitivity, individual character traits, and anxiety belong to second group. Patients with bruxism, both adults and children seem to present higher results on scales assessing the intensity of mental disorders, anxiety and stress when compared to the non-bruxers [3]. The third and getting more popular group of factors associated with bruxism are exogenous origin factors such as caffeine, nicotine, drugs, medicines and alcohol [3].

Sleep bruxism can affect approximately 13% of adult population [1,2,3]. The occurrence of sleep bruxism is the highest in childhood—14–20% and decreases with age [3,4,5]. Among teens, the occurrence of sleep bruxism is estimated at 12%, among adolescents at the level of 8%, and at the level of 3% in the elderly people [6,7,8]. The occurrence of this phenomenon is independent of gender and often family. It is estimated that approximately 20 to 50% of sleep bruxers have at least one member of the closest family presenting the same phenomenon. In addition, around 2/3 of the cases of sleep bruxism occurring in childhood also occurs in a form that persists in adult life [8]. The origin of bruxism is multifactorial [1,2,3]. There are also scientific reports indicating the comorbidity of bruxism with systemic disorders, such as: thyroid diseases, digestive system disorders, sleep disorders, or cardiovascular diseases [1,2,3,7,9,10]. Particularly important factors predisposing to the appearance of sleep bruxism include: personality type, genetic predisposition, taking certain medications, and the presence of stressful situations, using caffeine and nicotine (especially with regard to the influence of these substances on the sleep architecture).

There are many scientific reports indicating connection between sleep bruxism and stress. Winocur et al. in the study on self-reported bruxism associations with perceived stress reported that participants reporting sleep and awake bruxism showed higher scores of PSS-10 [11]. Also in the study performed by Abekura et al. in which stress was assessed first by measuring the participants’ salivary chromogranin A (CgA) levels and second, using a visual analog scale (VAS), findings suggest that there is a relationship between psychological stress sensitivity and sleep bruxism [12].

There are also scientific reports indicating a connection between the occurrence of bruxism and symptoms of depression. Gungormus et al. in the study assessing the relationship between anxiety, depression, and bruxism reported that the anxiety and depression results were statistically significantly higher for bruxers than non-bruxers [13]. However, the study was carried out on patients presenting symptoms of temporomandibular disorders, which may be of great importance in this case. Fernandes et al. tried to assess the relationship between sleep bruxism, painful temporomandibular disorders and psychoemotional status [14]. They reported that sleep bruxism seemed to be a risk factor for painful temporomandibular disorders, and this in turn was a risk factor for the occurrence of higher depression and non-specific physical symptoms levels. Exact cause-effect relationship could not be established.

Currently preferred, regarding bruxism as two separate phenomena described by two definitions created the need to study the contribution of etiological factors to both types of bruxism separately in order to determine their exact participation and possible differences. Because of the apparent complexity of the problem the aim of this study is to evaluate the possible correlation between occurrence of sleep bruxism, perceived stress, and depression symptoms.

## 2. Material and Methods

### 2.1. Participants

Participants group consisted of patients of the Prosthetic Dentistry Clinic functioning at the Department of Prosthetic Dentistry at the Wroclaw Medical University, Poland with probable sleep bruxism. Probable sleep bruxism was diagnosed based on the guidelines of the American Academy of Sleep Medicine, International Classification of Diseases–Ten–Clinical Modification (ICD-10-CM) [7,8].

The authors obtained the approved of the local Ethical Committee of the Wroclaw Medical University (ID KB-195/2017). Each study participant gave informed written consent. The Clinical Trial Registration information is: www.ClinicalTrials.gov (identifier NCT03083405).

### 2.2. Video-Polysomnography Participants’ Selection

Video-polysomnography was performed in selected patients. The selection was based on medical interview and physical examination. Researchers paid particular attention on sleep teeth grinding, preferred when confirmed by patients’ partner. Physical extra- and intraoral examination for a thorough reporting of the teeth and oral mucosa condition was performed for each patient. Attention was paid to the damage to the dental hard tissues (e.g., cracked teeth), mechanical wear of the teeth (i.e., attrition), indentations on the tongue and/or lip, linea alba on the inner cheek, masticatory muscle hypertrophy, repetitive failures of restorative work and prosthodontic constructions. The diagnosis of probable bruxism was based on the guidelines of ICD-10-CM and international consensus on the assessment of bruxism taking into account the present symptoms [1].

### 2.3. Exclusion Criteria

Participants were excluded from video-polysomnographic examination when they met the following criteria: lack of diagnosis of probable bruxism based on the ICD-10-CM criteria, severe systemic diseases (including severe mental diseases and disabilities), disorder that can cause sleep bruxism, intake of substances that significantly affect the function of the nervous and muscular system, inability to participate in polysomnography, lack of consent to take part in the study, age below 18 years.

### 2.4. Video-Polysomnographic Examination

Included patients were subjected to one-night video-polysomnography recorded with Nox A1 (Nox Medical, Reykjavik, Iceland) device. Examination took place in the Sleep Laboratory at the Wroclaw Medical University between 10.00 p.m. and 6.00 a.m. Patient’s preferences and sleeping habits were considered.

Polysomnographic examinations included electroencephalographic, electrocardiographic, electrooculographic, and electromyographic recording. The one exception from placing the electrodes in standard way recommended by a producer was placing bipolar electromyographic recording leads symmetrically on the both sides of the masseter muscles’ origin and insertion. Examination also included: recording of breathing activity based on abdominal and thoracic movements, audio and video recording and body position. The pulse, level of saturation, and plethysmographic data were recorded using NONIN WristOx2 3150 pulse oximeter (Nonin Medical, Inc., Plymouth, MN, USA). Full polysomnographic recording was performed using Noxturnal device (Nox Medical, Reykjavik, Iceland).

### 2.5. Bruxism Assessment

Bruxism assessment based on the ICD-10-CM guidelines. The electromyographic recording from the masseter muscles region and audio and video recordings have become the basis for qualifying the bruxism episodes. As the episodes of sleep bruxism we have qualified rhythmic activity of masseter muscles that occurred after a minimum of 3 s break from the last muscle activity. Episodes were often accompanied by specific movements in the orofacial region and grinding sounds. Bruxism Episodes Index (BEI) signifying the number of bruxism episodes per hour of sleep was used to assess bruxism intensity. Electromyographic pathways were qualified as phasic episodes lasting 0.25–2 s, tonic episodes lasting more than 2 s and mixed episodes.

The ICD-10-CM guidelines states that BEI value less than 2 indicates a lack of sleep bruxism, a value of 2–4 indicates mild and moderate sleep bruxism, while a value above 4 indicates severe sleep bruxism [7,8]. In accordance to the presented data the patients were then divided into study group—bruxers (BEI ≥ 2) and non-bruxers—control group (BEI < 2).

### 2.6. Perceived Stress Scale-10

Each patient was screened with and Perceived Stress Scale–10 (PSS-10) [15,16,17]. The PSS-10 questionnaire contains 10 questions about subjective feelings related to everyday problems, personal events, and behaviors induced by them and ways of coping over it in the last month. The scale is a simple and reliable test that can be useful both clinically and scientifically. The overall result is the sum of 10 individual questions. The maximum number of points to be obtained in the questionnaire is 40. The results from 0 to 13 points indicate a low exposure to stress, while results above 20–22 points indicate a high exposure to stress.

### 2.7. Beck’s Depression Inventory

Each of the patients was screened for symptoms of depression using Beck’s Depression Inventory (BDI) [18,19,20,21,22]. This is a questionnaire consisting of 21 questions about the symptoms of depression. Each question can be answered with one of four responses scored from 0 to 3 points. The maximum number of points that can be obtained is 63. Scores in the range of 1–10 points indicate a result that does not deviate from the norm. Scoring in the range of 11–16 points indicates mild mood disorders. The range of 17–20 points is located on the borderline of clinical depression, 21–30 points indicate moderate depression, 31–40 severe, and 41–63 for extreme depressive disorder. The scale is a simple and understandable screening tool.

### 2.8. Database

Data obtained from polysomnography and questionnaires served to create the database in Excel (Microsoft Corporation, Redmond, WA, USA). The selected elements of the database have been subjected to statistical analysis.

### 2.9. Data Analysis

During the statistical analysis, first, the use of parametric methods was preferred. Data were further transformed only if they did not fulfill the assumptions of the parametric methods. It could be for example caused by the distribution shapes. If the data after the transformation met the assumptions of the parametric methods, this type of analysis was performed. If the data after the transformation still did not meet the assumptions of the parametric methods, non-parametric methods were used for the analyzes, and the analyzes were performed on the original (untransformed) data.

In additional analyzes the division into a study and control group was used in accordance to the following scheme: BEI up to 2 (“<2”) and BEI 2 and above (“2+”), respectively. The preferred analytical approach to the study of differences between groups was the Student’s t-test for unrelated samples.

The data distributions shapes and deviations from the shape of the normal distribution were analyzed with the Shapiro–Wilk test.

The Statistica 13.1 (Statsoft, Cracow, Poland) program was used to analyze the obtained data. Statistically analyzed results were considered as significant when they appeared with a probability of *p* < 0.05.

## 3. Results

### 3.1. Sample Characteristics

A total of 77 patients were included in the study—56 women and 21 men. The participants of the polysomnography were all Caucasians, aged 18–63 (mean 34.8 ± 10.8). The study group consisted of 58 patients, and 19 patients were included to the control group, respectively.

### 3.2. Bruxism Episodes Index (BEI) Data Distribution

BEI data distribution deviated from the normal distribution (W = 0.8745, *p* < 0.0001). Before the analysis, BEI values underwent a logarithmic transformation. After that the data distribution did not differ significantly from the normal distribution (W = 0.9728, *p* = 0.10).

### 3.3. Bruxism Episodes Index (BEI) and Perceived Stress Scale–10 (PSS-10)

**Figure 1:**
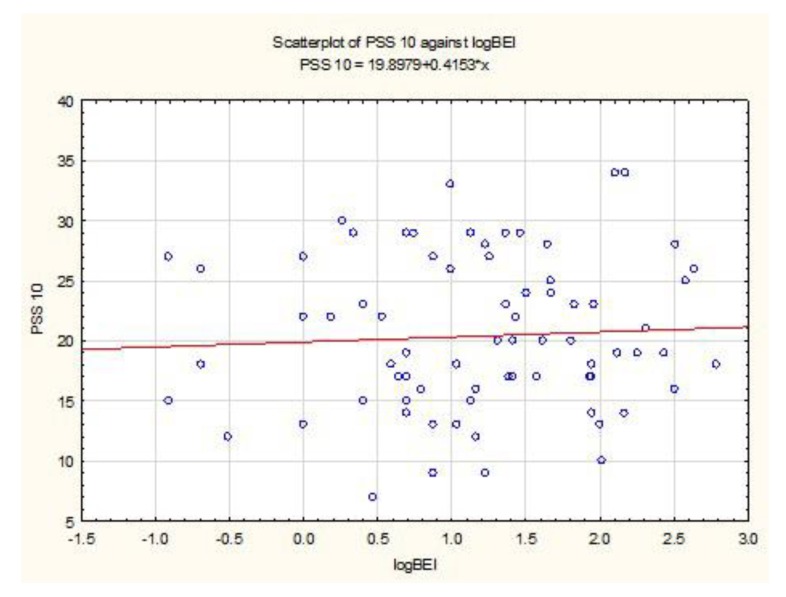
Relationship between Bruxism Episodes Index (BEI) and Perceived Stress Scale-10 (PSS-10) values.

The distribution of PSS-10 data did not differ from the normal distribution (W = 0.9767, *p* = 0.19). The Pearson’s linear correlation coefficient test was used to study the strength and significance of the log BEI relationship (BEI results after log transformation) and the PSS-10 result. The analysis showed no significant relationship between the BEI data after logarithmic transformation and the results of the PSS-10 questionnaire (r (74) = 0.06, *p* = 0.64) (Figure 1).

Descriptive statistics for PSS-10 values in relation to the studied and control groups are presented in Table 1.

The distribution of PSS-10 data in the control group did not differ from the normal distribution (W = 0.9654, *p* = 0.73). The distribution of PSS-10 data in the studied group did not differ significantly from the normal distribution (W = 0.9681, *p* = 0.14). Analysis of homogeneity of variance with the Leven test showed that the variances in the compared groups are homogeneous (F1,72 = 0.0004, *p* = 0.98).

**Figure 2:**
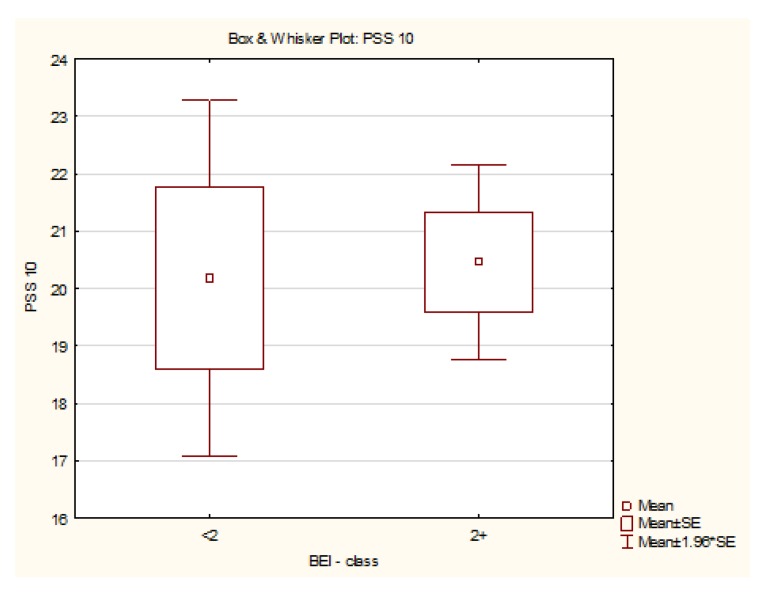
Summary of the PSS-10 scores for the studied and control group.

The data met the assumptions of the student’s t-test. The analysis showed no statistically significant difference in terms of PSS-10 between both groups (t72 = −0.16, *p* = 0.88) (Figure 2).

### 3.4. Bruxism Episodes Index (BEI) and Beck’s Depression Inventory (BDI)

**Figure 3:**
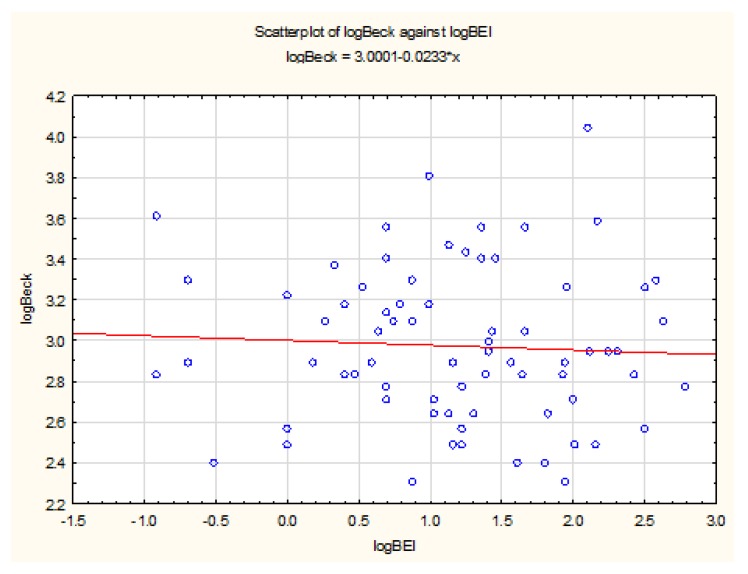
Relationship between BEI and Beck’s Depression Inventory (BDI) values.

The distribution of BDI data differed from the normal distribution (W = 0.8867, *p* < 0.0001). Before analysis, the values of the BDI questionnaire were subjected to a logarithmic transformation, after which there was no significant difference in this data from the shape of the normal distribution (W = 0.9792, *p* = 0.27). The Pearson’s linear correlation coefficient test was used to study the strength and significance of the relationship between logarithmic values of BEI and BDI. The analysis showed no statistically significant relationship between the BEI and BDI results after logarithmic transformation (r (73) = −0.05, *p* = 0.65) (Figure 3).

Descriptive statistics for Beck’s Depression Inventory are presented in Table 2.

**Figure 4:**
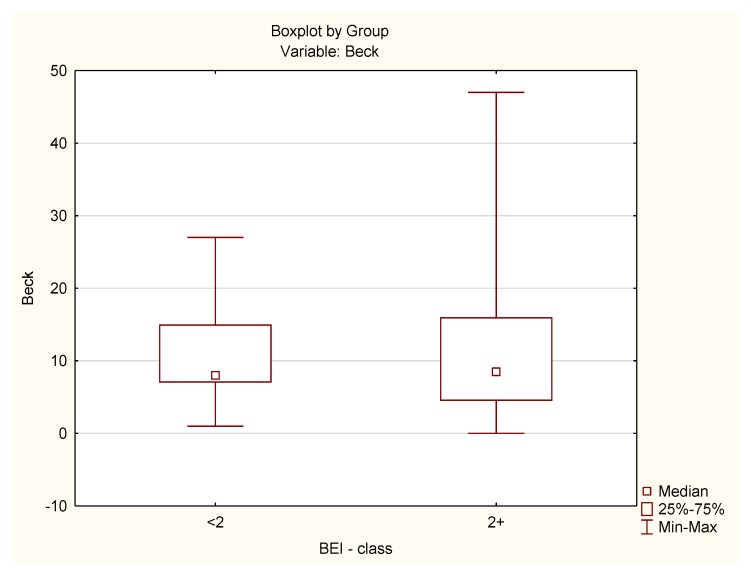
Summary of the BDI scores for the study and control groups.

The distribution of BDI data in the control group did not differ from the normal distribution (W = 0.9474, *p* = 0.42). The distribution of BDI data in the study group differed statistically significantly from the shape of the normal distribution (W = 0.8739, *p* = 0.00003). Because of the violation of the assumption about the normality of the data distribution in the study group, the analysis was carried out with the Mann–Whitney U test. The analysis showed that both groups did not differ statistically significantly in terms of BDI results (U = 453.5, *p* = 0.77) (Figure 4).

## 4. Discussion

The psychoemotional component is indicated as an important etiological factor for the occurrence of bruxism [1,2,3,9]. The main elements that belong to it are the severity of stress and anxiety [3]. The relationship between stress and sleep bruxism so far seemed to be so scientifically proven that it actually became the basis of the etiology of bruxism. There are a lot of studies indicating a positive correlation between stress and sleep bruxism relationship [23,24,25]. Ferreira-Bacci Ado et al. conducted a study which aimed to evaluate the behavior profile of a group of children diagnosed with bruxism [23]. Child Stress Scale was applied to measure the exposure to stress. The study findings suggested that behavioral problems and potential emotional problems can be risk factors to bruxism in children. Serra-Negra et al. in the study determining the relationship between stress levels, personality traits, and sleep bruxism in children reported that stress high levels are a key factor in sleep bruxism development among children [24]. In this case scientists also used Child Stress Scale. Similar findings among adults were obtained by Fluerașu et al. The study aimed to analyze the association between sleep bruxism, salivary cortisol, and psychological state in healthy adults. Scientists reported that subjects with sleep bruxism had a general status characterized by anxiety or stress compared to the control group [25]. The results of presented studies are limited because of the fact that the diagnosis of sleep bruxism was based on a questionnaire and intraoral examination and polysomnography was not performed. There are scientific studies that only partially support the hypothesis of the relationship between bruxism and stress [26,27,28]. Cavallo et al. in the study on the prevalence of awake and sleep bruxism and its correlation with perceived stress in a group of undergraduate students reported that correlation between stress and bruxism exists only for male gender [26]. Nakata et al. after the examination the relationship between psychosocial job stress and sleep bruxism also reported that sleep bruxism is only weakly associated with some aspects of job stress in men [27]. Furthermore, Muzalev et al. reported that psychological stress was a more important predictor factor for temporomandibular disorders pain than sleep bruxism [28]. There are also studies which do not support positive assertion between sleep bruxism and stress [29,30]. Pierce et al. in the study on 100 sleep bruxers reported no overall relationship between electromyographic measures and the personality variables nor between electromyographic measures and self-reported stress [29]. Also, Ohlmann et al. in the study aiming to identify associations between definite sleep bruxism and chronic stress and sleep quality reported that chronic stress and sleep quality do not seem to be associated with sleep bruxism [30].

The relationship between the occurrence of bruxism and depression is still considered controversial, because it has not been scientifically proven that depression can be the cause of the aggravation of bruxism. Some scientists, however, indicate a more frequent occurrence and greater severity of depressive symptoms in patients with bruxism [31,32]. This phenomenon can be explained in two ways. First of all, bruxism can be induced by some drugs used in the treatment of depression. Uca et al. reported an increased incidence of bruxism in patients taking antidepressants compared to the control group [31]. Second, taking into account bruxism as a risk factor for temporomandibular disorders, which often leads to the appearance or exacerbation of depressive symptoms through the reduction of quality of life. Fernandes et al. have shown that bruxism is a risk factor for temporomandibular disorders associated with pain, which indirectly also makes it a risk factor for depressive symptoms and non-specific psychological symptoms, but the direct causal relationship between bruxism and depression cannot be unambiguously confirmed [32].

The presented study attempts to investigate the relationship between the intensity of sleep bruxism assessed on the basis of BEI and psychoemotional condition assessed on the basis of the results of the Perceived Stress Scale–10 assessing stress exposure and Beck’s Depression Inventory questionnaire assessing the severity of depression symptoms. Statistical analysis showed that the increase in BEI does not correlate with the higher scores of the mentioned questionnaires, also taking into account the comparison between bruxers and non-bruxers. The interpretation of these data should take into account the fact that by 2017 all scientific consensus on the concept of bruxism presented a definition of this phenomenon that was universal for the awake and sleep bruxism [2,3]. Therefore, risk factors and clinical implications for both subtypes of bruxism were treated collectively, which prevented accurate assessment and possible differential diagnosis within the same, yet significantly different unit. As part of the 2018 international consensus, Lobbezoo et al. proposed the determination of sleep and awake bruxism with separate definitions [1]. New definitions may be the key to the modern interpretation of bruxism as two separate units, they may also explain the fact that there is a lack of dependency between sleep bruxism and both PSS-10 and BDI scores. Sleep bruxism really seems to be a different, independent unit that does not follow standard descriptions, the factors of which could be largely determined for awake and in this case they do not apply. The issue of splitting bruxism into two separate units undoubtedly requires a more thorough and broader comparative analysis also taking into account psychoemotional status concerning stress and depression.

Another important factor is the determination of individual participation of sleep bruxism and awake bruxism as risk factors for painful temporomandibular disorders, which in turn may lead to worsening of depression symptoms as Fernandes et al. have shown [32] and be related to greater stress exposure as Muzalev et al. have shown [28]. Temporomandibular disorders are being discussed as serious risk factors for anxiety and depression [33,34,35,36,37]. Furthermore, stress is being considered as a risk factor for development of temporomandibular disorders [28]. This four-way relationship between both types of bruxism, stress, depression, and temporomandibular disorders should be conducted to assess detailed causal relationships between them.

The differences between the number of participants of the study groups and the lack of evaluation of changes in psychological status and sleep bruxism in some period of time could be considered as the main limitations of the presented study. Despite the current ICD-10-CM guidelines that does not indicate the need for an adaptive night for sleep bruxism assessment states that: “First-night effect on RMMA index is minimal” [7,8], the only one-night polysomnography conducted in the presented study could also be potentially considered as the study limitation.

## 5. Conclusions

Intensity of sleep bruxism was not statistically significantly correlated with perceived stress based on PSS-10 and severity of depression based on BDI. The subject of the influence of the psychoemotional state on the severity of sleep bruxism should be further explored. There is a need for a comparative study of stress and depression in patients with sleep and awake bruxism also taking into account the occurrence of temporomandibular disorders. Given the results of the available literature, future analysis should be also carried out by comparing different age groups.

## Figures and Tables

**Table 1:** Descriptive statistics for PSS-10 values for studied and control group.

Group	Number	Mean	Median	Minimum	Maximum	Standard Deviation
studied group	57 *	20.46	19.00	9	34	6.519
control group	17 *	20.18	22.00	7	30	6.541

* Presented numbers differ from assumed number because of the cases of incorrect and incomplete PSS-10.

**Table 2:** Descriptive statistics for the Beck’s Depression Inventory divided into study and control groups.

Group	Number	Mean	Median	Minimum	Maximum	Standard Deviation
studied group	56 *	11.11	8.50	0	47	9.230
control group	17 *	10.71	8.00	1	27	6.734

* Presented numbers differ from assumed number because of the cases of incorrect and incomplete BDI.

## References

[1] Lobbezoo F., Ahlberg J., Raphael K.G., Wetselaar P., Glaros A.G., Kato T., Santiago V., Winocur E., De Laat A., De Leeuw R. (2018). International consensus on the assessment of bruxism: Report of a work in progress. J. Oral Rehabil..

[2] Manfredini D., Serra-Negra J., Carboncini F., Lobbezoo F. (2017). Current Concepts of Bruxism. Int. J. Prosthodont..

[3] Lobbezoo F., Ahlberg J., Glaros A.G., Kato T., Koyano K., Lavigne G.J., de Leeuw R., Manfredini D., Svensson P., Winocur E. (2013). Bruxism defined and graded: An international consensus. J. Oral Rehabil..

[4] Macedo C.R., Silva A.B., Machado M.A., Saconato H., Prado G.F. (2007). Occlusal splints for treating sleep bruxism (tooth grinding). Cochrane Database Syst. Rev..

[5] Castroflorio T., Bargellini A., Rossini G., Cugliari G., Rainoldi A., Deregibus A. (2015). Risk factors related to sleep bruxism in children: A systematic literature review. Arch. Oral Biol..

[6] Machado E., Dal-Fabbro C., Cunali P.A., Kaizer O.B. (2014). Prevalence of sleep bruxism in children: A systematic review. Dental Press J. Orthod..

[7] American Academy of Sleep Medicine (2014). International Classification of Sleep Disorders.

[8] World Health Organization International Classification of Diseases, Tenth Revision, Clinical Modification (ICD-10-CM). https://www.who.int/classifications/icd/icdonlineversions/en/.

[9] De Leeuw L.R., Klasser G.D. (2013). Orofacial Pain. Guidelines for Assessment, Diagnosis, and Management.

[10] Raphael K.G., Santiago V., Lobbezoo F. (2016). Is bruxism a disorder or a behavior? Rethinking the international consensus on defining and grading of bruxism. J. Oral Rehabil..

[11] Winocur E., Uziel N., Lisha T., Goldsmith C., Eli I. (2011). Self-reported bruxism—Associations with perceived stress, motivation for control, dental anxiety and gagging. J. Oral Rehabil..

[12] Abekura H., Tsuboi M., Okura T., Kagawa K., Sadamori S., Akagawa Y. (2011). Association between sleep bruxism and stress sensitivity in an experimental psychological stress task. Biomed. Res..

[13] Gungormus Z., Erciyas K. (2009). Evaluation of the relationship between anxiety and depression and bruxism. J. Int. Med. Res..

[14] Fernandes G., Franco A.L., Siqueira J.T., Gonçalves D.A., Camparis C.M. (2012). Sleep bruxism increases the risk for painful temporomandibular disorder, depression and non-specific physical symptoms. J. Oral Rehabil..

[15] Taylor J.M. (2015). Psychometric analysis of the Ten-Item Perceived Stress Scale. Psychol. Assess..

[16] Nielsen M.G., Ørnbøl E., Vestergaard M., Bech P., Larsen F.B., Lasgaard M., Christensen K.S. (2016). The construct validity of the Perceived Stress Scale. J. Psychosom. Res..

[17] González-Ramírez M.T., Rodríguez-Ayán M.N., Hernández R.L. (2013). The perceived stress scale (PSS): Normative data and factor structure for a large-scale sample in Mexico. Span. J. Psychol..

[18] Beck A.T., Ward C.H., Mendelson M., Mock J., Erbaugh J. (1961). An inventory for measuring depression. Arch. Gen. Psychiatry.

[19] Richter P., Werner J., Heerlein A., Kraus A., Sauer H. (1998). On the validity of the Beck Depression Inventory. A review. Psychopathology.

[20] Whisman M.A., Judd C.M., Whiteford N.T., Gelhorn H.L. (2013). Measurement invariance of the Beck Depression Inventory-Second Edition (BDI-II) across gender, race, and ethnicity in college students. Assessment.

[21] Robinson B.E., Kelley L. (1996). Concurrent validity of the Beck Depression Inventory as a measure of depression. Psychol. Rep..

[22] Piotrowski C. (1996). Use of the Beck Depression Inventory in clinical practice. Psychol. Rep..

[23] Ferreira-Bacci Ado V., Cardoso C.L., Díaz-Serrano K.V. (2012). Behavioral problems and emotional stress in children with bruxism. Braz. Dent. J..

[24] Serra-Negra J.M., Paiva S.M., Flores-Mendoza C.E., Ramos-Jorge M.L., Pordeus I.A. (2012). Association among stress, personality traits, and sleep bruxism in children. Pediatr. Dent..

[25] Fluerașu M.I., Bocsan I.C., Buduru S., Pop R.M., Vesa S.C., Zaharia A., Negucioiu M., Iacob S.M. (2019). The correlation between sleep bruxism, salivary cortisol, and psychological status in young, Caucasian healthy adults. Cranio.

[26] Cavallo P., Carpinelli L., Savarese G. (2016). Perceived stress and bruxism in university students. BMC Res. Notes.

[27] Nakata A., Takahashi M., Ikeda T., Hojou M., Araki S. (2008). Perceived psychosocial job stress and sleep bruxism among male and female workers. Community Dent. Oral Epidemiol..

[28] Muzalev K., Visscher C.M., Koutris M., Lobbezoo F. (2018). Long-term variability of sleep bruxism and psychological stress in patients with jaw-muscle pain: Report of two longitudinal clinical cases. J. Oral Rehabil..

[29] Pierce C.J., Chrisman K., Bennett M.E., Close J.M. (1995). Stress, anticipatory stress, and psychologic measures related to sleep bruxism. J. Orofac. Pain.

[30] Ohlmann B., Bömicke W., Habibi Y., Rammelsberg P., Schmitter M. (2018). Are there associations between sleep bruxism, chronic stress, and sleep quality?. J. Dent..

[31] Uca A.U., Uğuz F., Kozak H.H., Gümüş H., Aksoy F., Seyithanoğlu A., Kurt H.G. (2015). Antidepressant-Induced Sleep Bruxism: Prevalence, Incidence, and Related Factors. Clin. Neuropharmacol..

[32] Fernandes G., Siqueira J.T., Godoi Gonçalves D.A., Camparis C.M. (2014). Association between painful temporomandibular disorders, sleep bruxism and tinnitus. Braz. Oral Res..

[33] Nazeri M., Ghahrechahi H.R., Pourzare A., Abareghi F., Samiee-Rad S., Shabani M., Arjmand S., Abazarpour R. (2018). Role of anxiety and depression in association with migraine and myofascial pain temporomandibular disorder. Indian J. Dent. Res..

[34] Reiter S., Emodi-Perlman A., Goldsmith C., Friedman-Rubin P., Winocur E. (2015). Comorbidity between depression and anxiety in patients with temporomandibular disorders according to the research diagnostic criteria for temporomandibular disorders. J. Oral Facial Pain Headache..

[35] Dıraçoǧlu D., Yıldırım N.K., Saral İ., Özkan M., Karan A., Özkan S., Aksoy C. (2016). Temporomandibular dysfunction and risk factors for anxiety and depression. J. Back Musculoskelet Rehabil..

[36] Bertoli E., de Leeuw R. (2016). Prevalence of Suicidal Ideation, Depression, and Anxiety in Chronic Temporomandibular Disorder Patients. J. Oral Facial Pain Headache..

[37] Wieckiewicz M., Zietek M., Smardz J., Zenczak-Wieckiewicz D., Grychowska N. (2017). Mental Status as a Common Factor for Masticatory Muscle Pain: A Systematic Review. Front. Psychol..

